# TALEN-mediated shift of mitochondrial DNA heteroplasmy in MELAS-iPSCs with m.13513G>A mutation

**DOI:** 10.1038/s41598-017-15871-y

**Published:** 2017-11-14

**Authors:** Naoki Yahata, Yuji Matsumoto, Minoru Omi, Naoki Yamamoto, Ryuji Hata

**Affiliations:** 1 Department of Anatomy I, Fujita Health University School of Medicine, Toyoake, Aichi Japan; 2 Department of Pediatrics, Fujita Health University School of Medicine, Toyoake, Aichi Japan; 3 Division of Molecularbiology, Joint Research Support Promotion Facility, Center for Research Promotion and Support, Fujita Health University, Toyoake, Aichi Japan; 4 Department of Anatomy, Ehime University Graduate School of Medicine, Shitsukawa, To-on, Ehime Japan

## Abstract

Induced pluripotent stem cells (iPSCs) are suitable for studying mitochondrial diseases caused by mitochondrial DNA (mtDNA) mutations. Here, we generated iPSCs from a patient with mitochondrial myopathy, encephalopathy, lactic acidosis, and stroke-like episodes (MELAS) with the m.13513G>A mutation. The patient’s dermal fibroblasts were reprogrammed, and we established two iPSC clones with and without mutant mtDNA. Furthermore, we tried to decrease mutant mtDNA level in iPSCs using transcription activator-like effector nucleases (TALENs). We originally engineered platinum TALENs, which were transported into mitochondria, recognized the mtDNA sequence including the m.13513 position, and preferentially cleaved G13513A mutant mtDNA (G13513A-mpTALEN). The m.13513G>A heteroplasmy level in MELAS-iPSCs was decreased in the short term by transduction of G13513A-mpTALEN. Our data demonstrate that this mtDNA-targeted nuclease would be a powerful tool for changing the heteroplasmy level in heteroplasmic iPSCs, which could contribute to elucidation of the pathological mechanisms of mitochondrial diseases caused by mtDNA mutations.

## Introduction

Mitochondrial diseases are a heterogeneous group of multisystem disorders caused by mitochondrial dysfunction including defects of the respiratory chain/oxidative phosphorylation system. Some mitochondrial diseases are caused by a pathogenic mutation or deletion of mitochondrial DNA (mtDNA), which is an about 16.6kbp, double stranded, circular molecule encoding 37 genes, and has typically several thousand copies per cell in humans^1^. Many mitochondrial disease patients with mtDNA mutations possess both normal and mutant mtDNA in an individual cell, termed heteroplasmy. The degree of heteroplasmy and distribution of mutant mtDNA in the patient’s tissues determine the severity and phenotypic heterogeneity of this disease. Mitochondrial myopathy, encephalopathy, lactic acidosis and stroke-like episodes (MELAS) is one of the most common mitochondrial diseases caused by point mutations; m.3243A>G, m.3271T>C, m.13513G>A and others^2^. MELAS is predominantly characterized by fatal encephalopathy, manifested as seizures and/or stroke-like episodes^3^.

Because of the complex clinical symptoms including stroke-like episodes, disease models that reproduce cellular phenotypes in MELAS are eagerly awaited in order to develop radical treatment for this disease. Somatic cells can be directly reprogrammed using defined genetic factors to yield induced pluripotent stem cells (iPSCs), which have the capacity to differentiate into all types of somatic cells^4^. This technology allows us to create patient-specific pluripotent stem cells that retain the contents of the patient’s cells, including mtDNA. Here, we describe the establishment of iPSCs from a MELAS patient having a m.13513G>A mutation in mtDNA, to investigate the cell-specific effects of mitochondrial dysfunction and other disease phenotypes caused by mtDNA mutation.

In disease modeling using patient-derived iPSCs, to precisely evaluate the effect of a focused gene on cellular phenotypes, gene correction is necessary. Programmable nucleases, including zinc finger nuclease (ZFN), transcription activator-like effector nuclease (TALEN) and clustered regularly interspaced short palindromic repeats (CRISPR)/CRISPR associated protein 9 (Cas9), produce site-specific double-strand breaks (DSBs), which enhance the efficiency of targeted mutagenesis^5^. These nucleases (ZFN and TALEN) have recently shown the ability to alter mtDNA heteroplasmy level in patients’ cells or cybrids by introduction of DSBs into mtDNA with pathological mutations such as m.8344A>G^6^, m.8993T>G^7^, m.9176T>C^8^, m.13513G>A^6^ and m.14459G>A^8,9^. In this report, we demonstrated a shift of m.13513G>A heteroplasmy level in MELAS-iPSCs by TALEN.

TALENs are composed of a DNA binding domain (TALE) and a FokI nuclease domain. The DNA-binding specificity of TALE is provided by tandem copies of a 34 amino acid repeat unit, module, including DNA recognition code, ‘repeat-variable di-residue’ (RVD). Numerous methods of constructing TALENs harboring different TALE scaffolds and repeat variants have been reported^10^. We selected the Platinum Gate TALEN construction system as an efficient system for highly active platinum TALENs (pTALENs) with periodically-patterned repeat variants harboring non-RVD variations^11^. We originally designed and generated m.13513G>A mtDNA-targeted platinum TALEN (G13513A-mpTALEN) to recognize the mtDNA sequence including the m.13513 position and preferentially cleave mutant mtDNA (G13513A-mpTALEN). We demonstrated that the m.13513G>A heteroplasmy level in MELAS-iPSCs was decreased in the short term by transduction of G13513A-mpTALEN. Our mpTALEN system would be effective for changing the heteroplasmy level in iPSCs and could contribute to basic research on mitochondrial diseases caused by mtDNA mutations.

## Results

### Generation and characterization of iPSCs from MELAS patient with m.13513G>A mutation

A 16-year old male MELAS patient with the m.13513G>A mutation in mtDNA^12^ consented to skin biopsy. After expansion of primary dermal fibroblasts (named A01), cells were transduced with reprogramming factors by two methods. Fibroblasts were transduced with four transcription factors, *OCT3/4*, *SOX2*, *KLF4* and *c-MYC*, by Sendai virus. Alternatively, fibroblasts were transduced with six factors, *OCT3/4*, *SOX2*, *KLF4*, *L-MYC*, *LIN28*, and *p53*-shRNA, by episomal vectors, according to a previously reported procedure^13^. A few weeks later, a few dozen human embryonic stem cell (ESC)-like colonies emerged. We picked and expanded 33 and 47 iPSC colonies generated by Sendai virus and episomal vectors, respectively. Next, we determined the percentage of G13513A mutant mtDNA in iPSCs at passage (p) 5 by an allele refractory mutation system (ARMS)-based qPCR method (Figure S1). Most of the iPSC clones showed an undetectable level of mutant mtDNA; however, a few clones (#26, #30, #61, #67) showed the presence of mutant mtDNA (Figure S1). The #61-iPSC clone, which only possessed G13513A mutant mtDNA among all iPSC clones generated by Sendai virus (Figure S1), could not be expanded after passage 5. Therefore, we decided not to use any iPSC clones generated by Sendai virus for further analyses.

**Figure 1:**
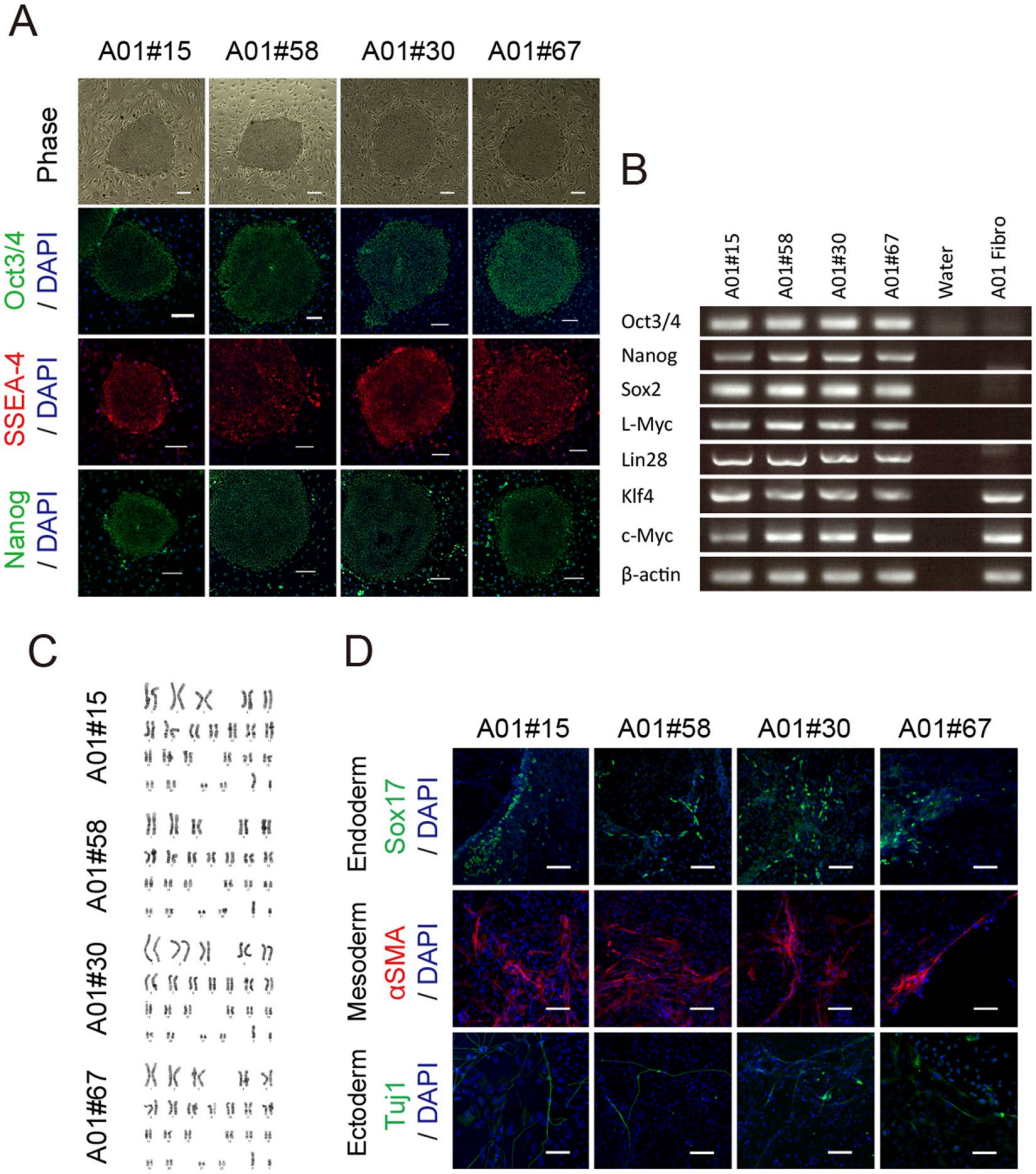
Establishment of human iPSCs from MELAS patient with m.13513G>A mutation. (**A**) Morphological features and expression of human ESC markers. All A01 MELAS-iPSC clones are identical to human ESCs. All iPSC clones expressed the human ESC markers Oct3/4 (green), SSEA-4 (red) and Nanog (green) analyzed by immunocytochemical analysis. Phase, phase-contrast image. Scale bar, 200 μm. (**B**) RT-PCR analyses of pluripotency markers and reprogramming factors (Oct3/4, Nanog, Sox2, L-Myc, Lin28, Klf4 and c-Myc). β-actin serves as a loading control. (**C**) Karyotype analysis revealed that all hiPSCs had a preserved normal karyotype. (**D**) *In vitro* differentiation of MELAS-iPSCs into all three germ layers. Sox17 (endoderm, green), αSMA (mesoderm, red) and Tuj1 (ectoderm, green). Scale bar, 100 μm.

Next, we selected a few integration-free MELAS-iPSC clones (#15, #58, #30, #67) which were confirmed by genomic PCR analyses (Figure S2). All of the selected iPSC clones showed characteristic human ESC-like morphological features (Fig. 1A). Immunocytochemical analysis revealed that these iPSC clones were Oct3/4, SSEA-4, Nanog positive (Fig. 1A). Reverse transcription-PCR (RT-PCR) analyses also showed that pluripotent markers such as *OCT3/4*, *NANOG*, *SOX2*, and *Lin28* were expressed in all iPSC clones and not in A01 fibroblasts (Figs 1B and S3). The pluripotency of these iPSC clones was confirmed by the presence of cell derivatives of all three germ layers in *in vitro* differentiation. Our selected iPSC clones formed embryoid bodies (EBs) in floating culture condition. After sixteen days of differentiation, Sox17- (endoderm), α-smooth muscle actin (α-SMA)- (mesoderm) and Tuj1- (ectoderm) positive cells were detected in each culture as previously reported (Fig. 1D) ^14^. Moreover, G-band analyses showed that all of these iPSC clones maintained a normal karyotype (Fig. 1C).

### Characterization of mtDNA in MELAS-iPSCs

**Figure 2:**
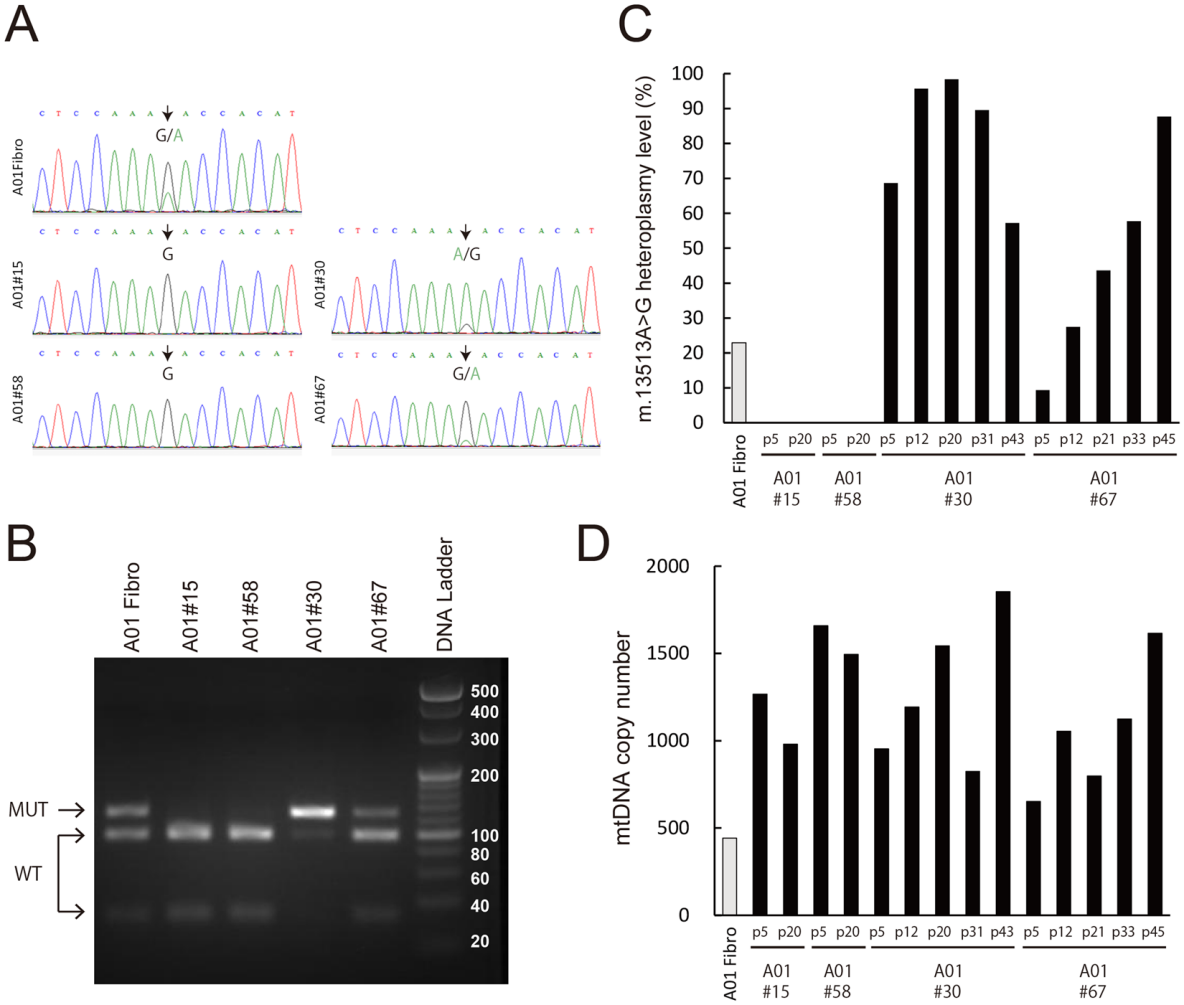
Characterization of mtDNA in A01 MELAS-iPSCs. (**A**) Genetic analysis of A01 fibroblasts and MELAS-iPSCs at passage 5 by Sanger sequencing. Arrows indicate m.13513 position. (**B**) RFLP analysis of A01 fibroblasts and MELAS-iPSCs. The 120 bp and 93 bp + 27 bp fragments indicate the presence of mutant and wild-type mtDNA, respectively. Heteroplasmy level of m.13513G>A mtDNA (**C**) analyzed by ARMS-qPCR and mtDNA copy number (**D**) in A01 fibroblasts and MELAS-iPSCs. MELAS-iPSCs were collected at the following passage numbers: #15 at p5 and p20; #58 at p5 and p20; #30 at p5, p12, p20, p31 and p43; #67 at p5, p12, p21, p33 and p45.

The presence and percentage of mutant mtDNA level in the MELAS patient-derived dermal fibroblasts (A01 fibroblasts) and selected iPSC clones (#15, #58, #30, #67) at passage 5 were re-evaluated by Sanger-sequencing (Fig. 2A) and PCR-RFLP (Fig. 2B). These data and ARMS-qPCR analysis (Figs 2C and S1) showed a similar trend in the percentage of G13513A mutant mtDNA. G13513A mutant mtDNA was undetected in two iPSC clones, #15 and #58, at passage 5 (Figs 2A,B,C and S1) as well as passage 20 (Fig. 2C). In contrast, the other clones, #30 and #67, showed the presence of G13513A mtDNA at passage 5 (Figs 2A,B,C and S1). Furthermore, we serially examined m.13513G>A heteroplasmy level in these clones, which fluctuated during cultivation (Figs 2C and S4A).

mtDNA copy number per cell in established MELAS-iPSC clones and A01 fibroblasts was determined by quantitative real-time PCR method (Fig. 2D). Our data showed that mtDNA copy number of MELAS-iPSCs ranged from 652 (#67, p5) to 1854 (#30, p43), which fluctuated throughout long-term cultivation (Fig. 2D). These data also indicate that mtDNA copy number did not correlate well with heteroplasmy level.

### Generation and characterization of G13513A-mpTALEN

**Figure 3:**
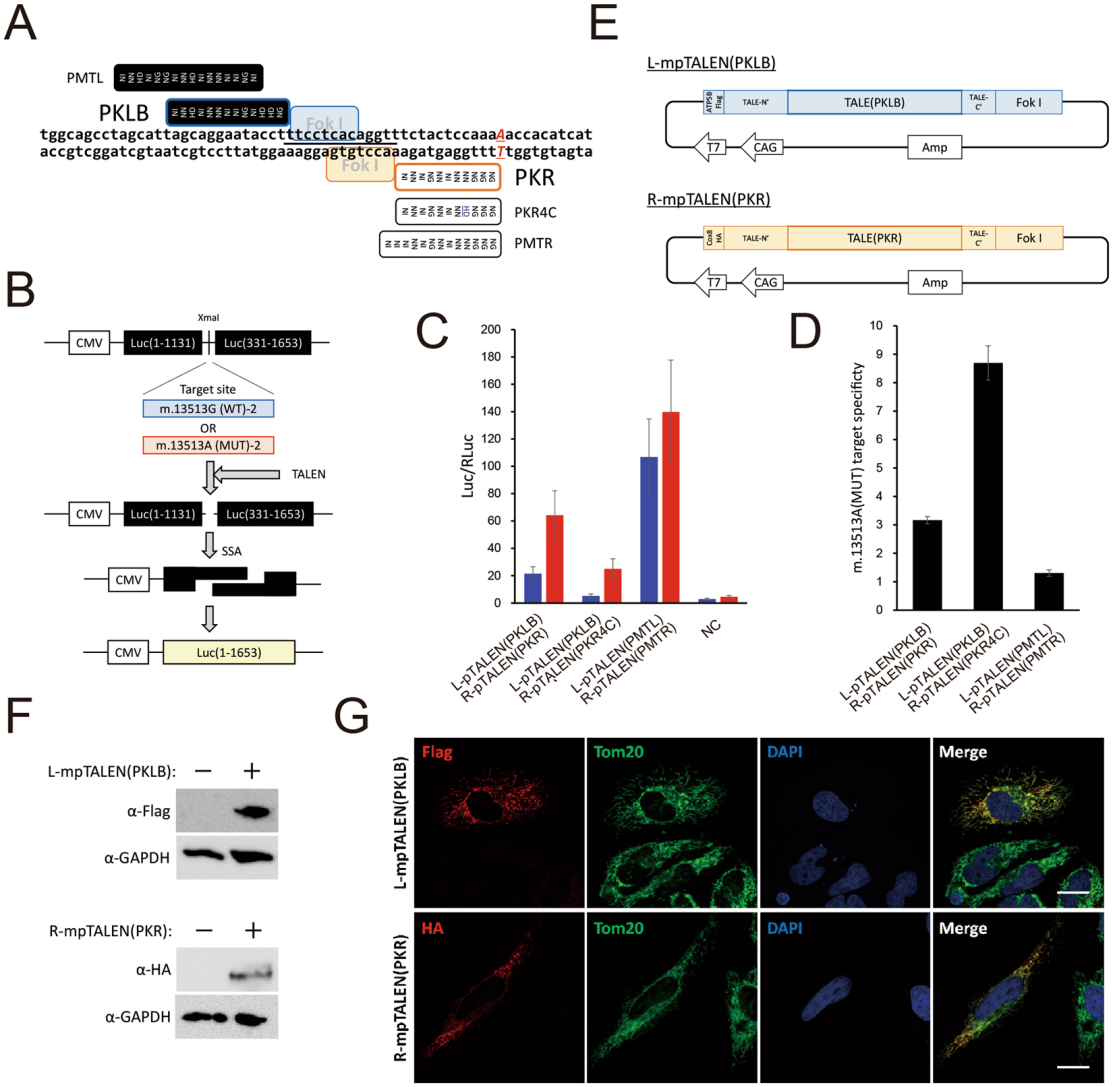
Generation of G13513A-mpTALEN. (**A**) Schematic illustration of target site of G13513A-pTALEN. Black and white boxes indicate RVDs of L-pTALEN and R-pTALEN, respectively. Letters beside box indicate TALE’s name. Black bar indicates spacer region of L-pTALEN(PKLB)/R-pTALEN(PKR) pair. (**B**) Scheme of SSA assay. The reporter plasmid encodes a part of the mtDNA sequence including m.13513G or m.13513A, named m.13513 G(WT)-2 or m.13513A(MUT)-2, which is sandwiched by two split inactive parts of the luciferase gene with overlapping repeated sequences. Following a double strand break caused by TALENs, a functional luciferase gene is generated by an SSA reaction. (**C**) Evaluation of SSA activity (Luc/RLuc) of pTALEN pairs. Blue and red bars reflect cleaving activity against m.13513 G(WT)-2 and m.13513A(MUT)-2, respectively. NC, negative control. Data are expressed as mean SEM (n = 3). (**D**) m.13513A(MUT) target specificity of each L-pTALEN/R-pTALEN pair. Data are expressed as mean SEM (n = 3). (**E**) Components of plasmids used to express L-mpTALEN(PKLB) and R-mpTALEN(PKR) monomers. (**F**) Western blotting of lysed HEK293T cells transfected with plasmid coding L-mpTALEN(PKLB) or R-mpTALEN(PKR) for 2 days. L-mpTALEN(PKLB) or R-mpTALEN(PKR) was detected using anti-Flag or anti-HA antibodies, respectively. GAPDH serves as a loading control. (**G**) Intracellular localization of mpTALENs analyzed by immunocytochemical analysis. L-mpTALEN(PKLB) or R-mpTALEN (PKR) was transiently expressed in HeLa cells. Two days after transfection, L-mpTALEN(PKLB) or R-mpTALEN(PKR) was stained using anti-Flag or anti-HA antibodies, respectively (red). Mitochondria were stained using anti-TOM20 antibodies (green). Nuclei were stained with DAPI (blue). Scale bar, 20 μm.

Recent reports showed that mtDNA-targeted site-specific nucleases, mtZFN and mitoTALEN, have the ability to alter mtDNA heteroplasmy level in living cells^6–9,15^. In this report, we tried to change the mutation load of m.13513G>A mtDNA in heteroplasmic iPSCs by TALEN. We originally established a mtDNA-targeted platinum TALEN (mpTALEN) expression system controlled by the CAG promoter (Fig. 3E). The m.13513G>A mtDNA-targeted platinum TALEN (G13513A-mpTALEN) means that the mpTALEN would recognize m.13513G>A mutant mtDNA (Fig. 3A) and preferentially induce DSBs in mutant mtDNA.

Because the FokI domains need to dimerize to digest DNA, two TALEN monomers (L- and R-pTALENs) are required to bind adjacent sequences on opposing strands. Firstly, we designed various types of G13513A-pTALEN pairs, which satisfied the following condition: immediately upstream of the DNA sequence recognized by RVDs of pTALEN monomer (position 0) is “T” (Figs 3A and S5). We constructed L- and R-pTALEN plasmids according to the method established by Yamamoto and colleagues^11^. Assembly of a custom pTALEN construct involves two steps: (i) four modules are ligated into intermediate array vectors and (ii) constructed repeat arrays are joined into a final expression vector. To examine the activity and specificity of the designed G13513A-pTALEN, we performed mammalian cell-based single-strand annealing (SSA) assay^16^ using a reporter plasmid having a part of the mtDNA sequence including m.13513G (WT) or m.13513A (MUT) (Figs 3B and S5A).

Wild-type (m.13513G) or mutant (m.13513A) mtDNA cleaving activity (Luc/RLuc) was calculated from dual luciferase measurements (Figs 3C and S5). The m.13513A (MUT) target specificity of each L-pTALEN and R-pTALEN pair was calculated as [Luc/RLuc(MUT, pTALEN + ) – Luc/RLuc(MUT, NC)] / [Luc/RLuc(WT, pTALEN + ) – Luc/RLuc(WT, NC)] (Fig. 3D). Most of the designed G13513A-pTALEN pairs showed low cleaving activity or low specificity for m.13513G>A mutant mtDNA (Figure S5B,C and D). On the other hand, we found one G13513A-pTALEN pair (L-pTALEN(PKLB) with 13 RVDs and R-pTALEN(PKR) with 11 RVDs), which showed relatively high activity and specificity for the G13513A mtDNA sequence (Fig. 3A,C and D). Furthermore, R-pTALEN(PKR) was modified to increase m.13513A (MUT) target specificity. The RVD at position 4 of the R-pTALEN(PKR) monomer was changed from “NN” to “HD”, named R-pTALEN(PKR4C). The m.13513A(MUT) target specificity and cleaving activity of the L-pTALEN(PKLB) and R-pTALEN(PKR4C) pair are higher and lower than those of the L-pTALEN(PKLB) and R-pTALEN(PKR) pair, respectively (Fig. 3C and D). We also analyzed L-pTALEN(PMTL) and R-pTALEN(PMTR) pair, generated based on the RVDs of m.13513G>A mitoTALEN^6^ (Fig. 3A). This pTALEN pair showed the highest cleaving activity (Fig. 3C), but the lowest m.13513G>A target specificity among the three pTALEN pairs (Fig. 3D).

Next, G13513A-pTALEN was modified to localize and function in mitochondria (G13513A-mpTALEN). The left- or right-mpTALEN monomer (L-mpTALEN or R-mpTALEN) has a mitochondrial targeting sequence (MTS) of ATP5B^7^ or Cox8^17^ and an epitope tag, Flag or HA at the N terminus, respectively (Figs 3E and S6). Western blot analysis showed that L-mpTALEN(PKLB) or R-mpTALEN(PKR) protein expressed in HEK293T cells by transient transfection was detected by Flag or HA tag specific antibodies, respectively (Fig. 3F).

In order to examine whether mpTALENs are readily imported into mitochondria, we analyzed their cellular localization in HeLa cells by their transient expression. Immunofluorescence analysis revealed that mpTALENs co-localized with the mitochondrial marker, Tom20, and were not observed in the nucleus (Figs 3G and S7).

### G13513A-mpTALEN increased heteroplasmy level in MELAS-iPSCs

**Figure 4:**
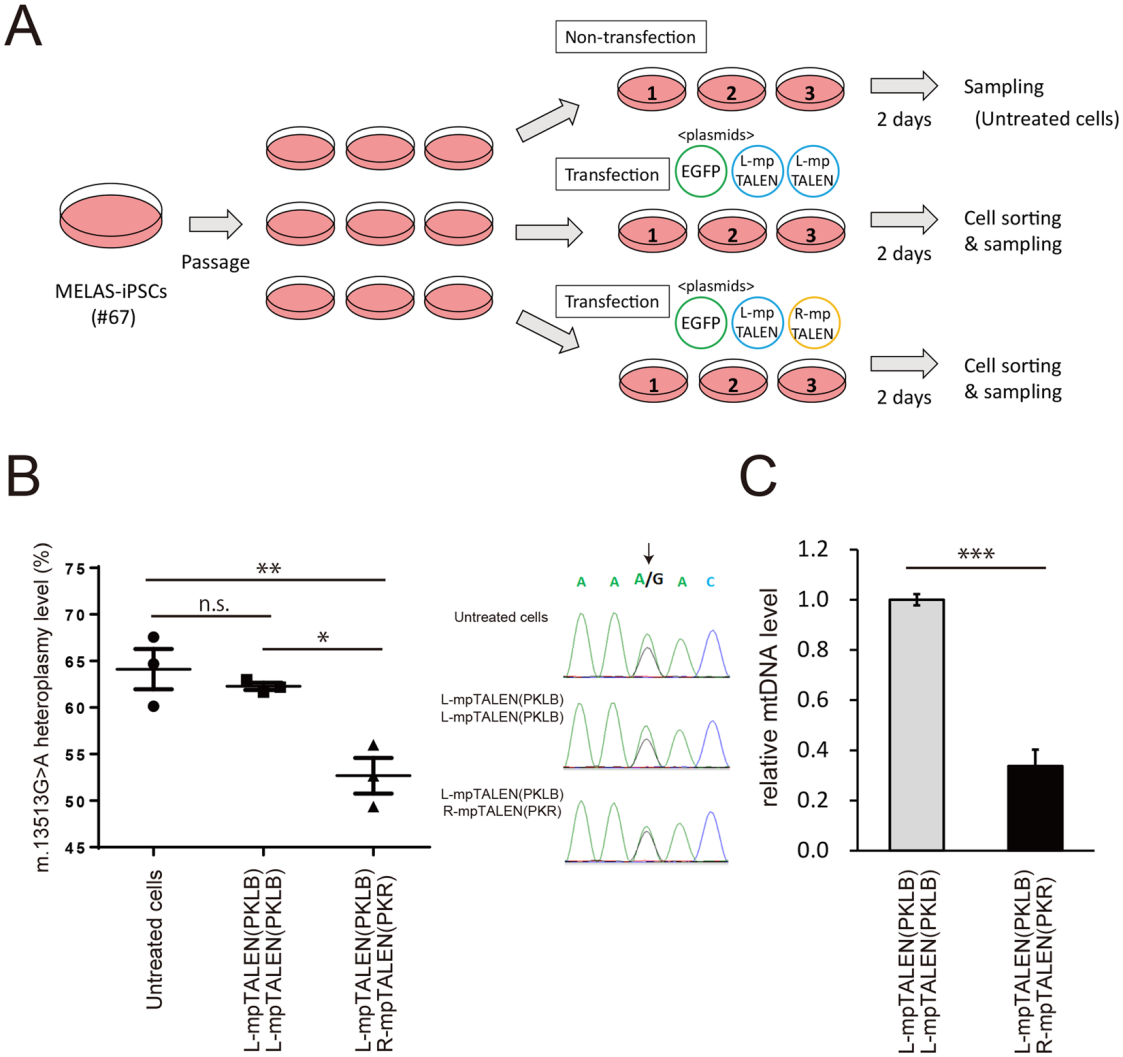
Decrease of m.13513G>A heteroplasmy level in MELAS-iPSCs by G13513A-mpTALEN. (**A**) Experimental scheme. MELAS-iPSCs (#67) subcultured at the same time were transfected with plasmids coding L-mpTALEN(PKLB) and R-mpTALEN(PKR) and EGFP (n = 3). EGFP-positive and live cells sorted at day 2 after transfection, named L-mpTALEN(PKLB)/R-mpTALEN(PKR), were compared with sorted cells transfected with twice the amount of plasmid coding L-mpTALEN(PKLB), named L-mpTALEN(PKLB)/L-mpTALEN(PKLB), and untreated cells. (**B**; left) m.13513G>A heteroplasmy level was analyzed by ARMS-qPCR. Data are expressed as mean ± SEM (n = 3). *p < 0.05, **p < 0.01, Tukey’s test. (**B**; right) Sanger-sequence analysis. Arrow indicates m.13513 position. (**C**) MtDNA copy numbers of L-mpTALEN(PKLB)/R-mpTALEN(PKR) are represented relative to those of L-mpTALEN(PKLB)/L-mpTALEN(PKLB). Data are expressed as mean ± SEM (n = 3). ***p < 0.005, Student’s *t*-test.

We next tried to alter mtDNA heteroplasmy level in MELAS-iPSCs using three types of G13513A-mpTALEN pairs, (i) L-mpTALEN(PKLB) and R-mpTALEN(PKR), (ii) L-mpTALEN(PKLB) and R-mpTALEN(PKR4C), and (iii) L-mpTALEN(PMTL) and R-mpTALEN(PMTR) (Fig. 3A). G13513A-mpTALEN plasmids were introduced into #67 MELAS-iPSCs cultured in feeder-free conditions by lipofection. EGFP was co-expressed as a marker for the transformant. At 1 or 2 days after transfection, EGFP-positive and live (propidium iodide-negative) cells were sorted using a cell sorter (Figure S8). m.13513G>A heteroplasmy level was analyzed in sorted cells, compared with that in untreated cells or sorted cells transfected with twice the amount of L-mpTALEN plasmid, so as not to change the total concentration of plasmids and expression levels of unnatural proteins. Each experiment was performed using heteroplasmic iPSCs subcultured at the same time (Figs 4A and S9A). ARMS-qPCR data showed that expression of L-mpTALEN/R-mpTALEN or L-mpTALEN for 1 day decreased the m.13513G>A mutation load in heteroplasmic iPSCs compared with that in untreated cells (Figure S9B,C and D). The L-mpTALEN(PKLB)/R-mpTALEN(PKR) pair showed the greatest reduction of mutation load (Figure S9B). The difference in m.13513G>A mutation load between sorted cells expressing L-mpTALEN(PKLB)/R-mpTALEN(PKR) and those expressing only L-mpTALEN(PKLB) for 1 day was also greatest (10.8%) among the three mpTALEN pairs, but was not significant (*p* = 0.0527; Tukey’s test) (Figure S9B). A decrease of mtDNA copy number caused by nuclease activity of the L- and R-mpTALEN pair was observed (Figure S9E,F and G). However, the effect caused by application of L-mpTALEN(PKLB)/R-mpTALEN(PKR4C) was not significant (Figure S9F), consistent with the SSA assay data showing that mtDNA cleaving activity of L-pTALEN(PKLB)/R-pTALEN(PKR4C) pair was lowest among the three pTALEN pairs (Fig. 3C).

The effect of the most hopeful L-mpTALEN(PKLB)/R-mpTALEN(PKR) pair on the m.13513G>A mutation load in #67-iPSCs at day 2 after transfection was also analyzed (Fig. 4A). The percentage of G13513A mutant mtDNA in sorted cells expressing the L-mpTALEN(PKLB) and R-mpTALEN(PKR) pair, sorted cells expressing only L-mpTALEN(PKLB), and untreated cells was 52.7%, 62.3% and 64.1%, respectively (Fig. 4B). The Sanger-sequencing data also showed that expression of both L-mpTALEN(PKLB) and R-mpTALEN(PKR) induced a significant reduction of the percentage of mutant mtDNA compared with the other samples (Fig. 4B). Copy number of mtDNA in sorted cells expressing L-mpTALEN(PKLB)/R-mpTALEN(PKR) at day 2 after transfection was decreased by 66% compared with that in sorted cells expressing only L-mpTALEN(PKLB) (Fig. 4C). Another two independent experiments also showed a similar tendency (Figure S10). Additionally, the effect of this G13513A-mpTALEN expression for 3 days on the mutation load in #67-iPSCs (Figure S11) was similar to that for 2 days. These data demonstrate that G13513A-mpTALEN could decrease m.13513G>A heteroplasmy level in MELAS-iPSCs.

## Discussion

In this study, we could obtain MELAS-iPSC clones both with and without G13513A mutant mtDNA by reprogramming MELAS patient’s fibroblasts. All MELAS-iPSC clones share the same nuclear genetic background, which gives us the chance to precisely analyze phenotypic differences in various types of differentiated cells with and without mutant mtDNA. To date, disease-specific iPSCs have been established for mitochondrial diseases caused by mtDNA mutations including MELAS^18–26^. The dominant mutation in MELAS, m.3243A>G in the *transfer RNA Leucine* ^(*URR*)^ gene, results in various types of mitochondrial respiratory chain deficiency in different patients. In some reports, biochemical analysis of biopsy samples and iPSC-derived cells with the m.3243A>G mutation showed deficiency of respiratory complex I^21^. Additionally, the m.13513G>A mutation in the *ND5* gene mainly induced a decrease of complex I activity^27^. To understand the pathological mechanism of MELAS, it is significant to identify the cellular phenotype caused by complex I deficiency using MELAS-iPSCs with the m.13513G>A mutation.

We reprogramed a MELAS patient’s fibroblasts by two methods. In the cloning process of iPSCs, there was no clear difference between Sendai virus-reprogramming and episomal vector-reprogramming. A heteroplasmic iPSC clone only generated by Sendai virus-reprogramming, #61, could not be expanded. The #61 clone showed the highest heteroplasmy level at passage 5 among all clones (Figure S1). One of our speculations about the cause of expansion failure is that the change of energy metabolism including the decrease of electron transfer complex I activity caused by a high mutation load of m.13513G>A inhibits cell proliferation and/or perfect reprogramming.

In this report, we originally established a mtDNA-targeted platinum TALEN, named mpTALEN, controlled by the CAG promoter for application to various types of cells including iPSCs. (Fig. 3E). Previous reports showed that mitochondrial site-specific nucleases (mitoTALEN and mtZFN) designed to target point mutations in mtDNA could decrease the percentage of mutant mtDNA in such as cybrids^6–9^. TALEN is the most selectable tool to target various types of point mutations in mtDNA because TALEN has multiple options of DNA recognition motif (TALE) for the target sequence and can be transported into mitochondria by addition of MTS. Otherwise, CRISPR/Cas9 is a simple and widespread system to cleave a targeted sequence selected by a guide RNA, but it is unclear whether the guide RNA component is transported into mitochondria. ZFN could not recognize every nucleotide codon although the DNA recognition motif in ZFN (zinc finger motif) is relatively shorter.

We here report that mpTALEN shifted the percentage of mutant mtDNA in MELAS-iPSCs. In the SSA assay, the L-pTALEN(PKLB)/R-pTALEN(PKR) pair showed both high cleaving activity and high m.13513A (MUT) target specificity, but the L-pTALEN(PKLB)/R-pTALEN(PKR4C) and L-pTALEN(PMTL)/R-pTALEN(PMTR) pairs showed low cleaving activity and low m.13513A (MUT) target specificity, respectively (Fig. 3C and D). Expression of the L-mpTALEN(PKLB)/R-mpTALEN(PKR) pair for 2 days was the most effective for inducing an about 10% decrease in m.13513G>A heteroplasmy level in MELAS-iPSCs (#67) (Fig. 4A). On the other hand, L-mpTALEN(PMTL)/R-mpTALEN(PMTR) pair, generated based on the RVDs of m.13513G>A mitoTALEN^6^, was not as effective as L-mpTALEN(PKLB)/R-mpTALEN(PKR) pair (Figure S9B and D). These data suggest that suitable RVDs of mpTALEN pair for G13513A mtDNA do not necessarily correspond to those of mitoTALEN pair because amino acid sequence of mpTALEN including non-RVD variations in modules is different from that of mitoTALEN. Our data also indicate that it is effective to select an mpTALEN pair for changing the mutation load in heteroplasmic iPSCs based on two values (cleaving activity and target specificity) calculated from SSA assay data. Otherwise, the heteroplasmy level in MELAS-iPSCs fluctuated during long-term cultivation (Figure S4). For example, the heteroplasmy level in #67-iPSCs decreased from 67.0% to 61.4% during 8-day cultivation under feeder-free conditions (Figure S4B). These data demonstrate the infeasibility of achieving an about 10% decrease in heteroplasmy level by normal cultivation for 2 days and the effectiveness of G13513A-mpTALEN in decreasing the m.13513G>A mutation load in heteroplasmic iPSCs in the short term.

Heteroplasmy shifts by mtDNA-targeted nuclease were accompanied by a decrease of mtDNA copy number, although transient mtDNA elimination recovered through uncharacterized mechanisms^28^. However, a marked decrease in mtDNA copy number potentially induces cellular toxicity^29,30^. The SSA assay data showed the L-pTALEN(PKLB)/R-pTALEN(PKR) pair has a little cleaving activity against wild-type mtDNA (Fig. 3C). It is necessary to develop more effective G13513A-mpTALEN to decrease the heteroplasmy level and to cleave wild-type mtDNA as little as possible in heteroplasmic cells. Additionally, expression of the L-mpTALEN monomer in #67-iPSCs for 1 day induced a significant decrease in m.13513G>A heteroplasmy level (Figure S9), but not for 2 days (Figs 4C and S10). This heteroplasmy shift shortly after transfection might be caused by the lipofection process. Further research about the effects of reported mtDNA-targeted nucleases^6–9^ and our G13513A-mpTALEN on various types of heteroplasmic cells might provide hints about the complicated mechanism controlling the heteroplasmy level.

In mitochondrial diseases with mtDNA mutations, understanding the relationship between clinical phenotypes and the level of mutant mtDNA in biopsy samples such as muscle^31^ is necessary as a guide to prognosis. Furthermore, elucidation of the relationship between heteroplasmy level and cellular phenotype is important to understand the pathological mechanism and develop therapies including drug discovery. We could get heteroplasmic iPSCs with high mutation loads by long-term culture on feeder layer (Figs 2C and S4A). Furthermore, we demonstrated a decrease of mutation load in heteroplasmic iPSCs by G13513A-mpTALEN in the short term (Figs 4B and S10). Phenotypical analyses using MELAS-iPSCs possessing a variety of mutation loads would contribute to elucidation of disease mechanisms and the pathological threshold in disease-relevant cells.

In summary, we generated MELAS-iPSCs from a patient possessing the G13513A mutation in mtDNA. We established two iPSC clones with and without mutant mtDNA. We showed that mpTALEN could decrease the percentage of mutant mtDNA in MELAS-iPSCs. These iPSCs and mpTALEN could contribute to investigation of the pathogenic mechanisms and therapeutic development for mitochondrial diseases with mtDNA mutations including MELAS.

## Methods

### Human iPSC generation and culture

We obtained a skin biopsy specimen from a MELAS patient^12^. This study was approved by the Ethics Committee of Ehime University and Fujita Health University, and informed consent was obtained from the patient’s guardian in accordance with the Declaration of Helsinki. We expanded the patient’s fibroblasts (named A01) in DMEM containing 10% FBS and 1% antibiotic-antimycotic solution (Sigma). Generation of iPSCs was performed by two methods. (1) A01 fibroblasts were transduced with four reprograming factors (*OCT3/4*, *SOX2*, *KLF4*, *c-MYC*) using CytoTune-iPS ver. 1.0 (DANAVEC) according to manufacturer’s protocol. (2) A01 fibroblasts were transduced with six reprogramming factors (*SOX2*, *KLF4*, *OCT3/4*, *L-MYC*, *LIN28*, *p53*-shRNA) by episomal vectors (pCXLE-hOCT3/4-shp53-F, Addgene #27077; pCXLE-hSK, #27078; and pCXLE-hUL, #27080, donated by Shinya Yamanaka)^13^ using Nucleofector^TM^ 2b (Lonza) with an Amaxa Human Dermal Fibroblast Nucleofector Kit (Lonza). Seven days after transduction, fibroblasts were plated on a mitomycin C-treated SNL feeder layer. The next day, the medium was changed to primate embryonic stem cell medium (ReproCELL, Japan) supplemented with basic fibroblast growth factor (bFGF) (Wako, Japan). After a few weeks, ESC-like colonies were mechanically dissociated and transferred onto new plates. iPSCs were harvested by treatment with CTK solution consisting of 0.1 mg/mL collagenase IV (Gibco), 0.25% trypsin (Gibco), 0.1 mM CaCl_2_ (Wako) and 20% KSR (Gibco).

### RNA isolation and reverse transcription (RT)-PCR

Total RNA was purified using an RNeasy Plus Mini Kit (Qiagen). One microgram of total RNA was used for reverse transcription reaction with ReverTra Ace and primer mix (Toyobo, Japan), according to the manufacturer’s protocol. PCR was performed with Ex Taq (TAKARA, Japan). PCR cycle conditions consisted of 95 °C-5 min, 30 cycles of 95 °C-20 sec and 60 °C-30 sec, and final cooling to 4 °C. Primer sequences are shown in Table S1. PCR products were run on 1% agarose gel and stained with ethidium bromide.

### Karyotyping analysis

Standard G-band chromosome analysis was performed by Nippon Gene Research Laboratories, Inc. (Miyagi, Japan).

### *In vitro* differentiation of iPSCs

iPSC clumps in DMEM/F12 (Gibco) containing 20% KSR (Gibco), 2 mM GlutaMax (Gibco), 1 × 10^−4^ M NEAA (Gibco), 1 × 10^−4^ M 2-ME (Sigma), and 1 × antibiotic and antimycotic solution (Sigma) were transferred to petri dishes. After 8-day floating culture, embryoid bodies (EBs) were transferred onto ECL (Millipore)-coated slide glasses, and incubated for another 8 days. After incubation, the cells were fixed with 4% paraformaldehyde in PBS.

### Immunocytochemical analysis

Cells were fixed with 4% paraformaldehyde/PBS for 30 min and incubated in PBS containing 0.2% Triton X-100 for 10 min. After blocking with 2% BSA/PBS for 1 h, cells were incubated with primary antibodies diluted with blocking buffer and then washed with PBS. Finally the cells were incubated with secondary antibodies, washed with PBS, and mounted using ProLong Diamond Antifade Mountant with DAPI (Molecular Probes). Immunoreactive cells were visualized using an LSM710 Laser Scanning Microscope (Carl Zeiss) or Biorevo BZ-9000 fluorescence microscope (Keyence).

### DNA isolation

Genomic DNA including mitochondrial DNA was isolated from fibroblasts and iPSCs using a QIAamp DNA Mini Kit (Qiagen) or NucleoSpin Tissue XS (Macherey-Nagel) according to their manufacturers’ protocols.

### Analysis of mtDNA mutation by DNA sequencing

A mitochondrial genome fragment including the m.13513 position was amplified using PrimeSTAR GXL DNA polymerase (TAKARA) with PCR primers (Mito-3F and Mito-3R, described in Table S1). The PCR amplicon was purified using Wizard SV Gel and PCR Clean-UP System (Promega) and sequenced using ARMS_G13513_R1 primer (Table S1) by a sequencing service (FASMAC Co., Ltd., Kanagawa, Japan).

### Analysis of mtDNA heteroplasmy

We analyzed the m.13513G>A mtDNA heteroplasmy level in fibroblasts and iPSCs by two methods. Restriction fragment length polymorphism (RFLP) analysis of the G13513A mutation was performed according to a previously reported protocol^27^, with partial modification. The 120-bp fragment amplified by PCR using the G13513A.02F and G13513A.02R primers (described in Table S1) was digested with a restriction enzyme, Bpi I (Thermo Scientific), run on 4% agarose gel and stained with ethidium bromide, resulting in 93-bp and 27-bp bands corresponding to the WT sequence. PCR cycle conditions consisted of 94 °C-1 min, 35 cycles of 94 °C-30 sec, 55 °C-30 sec, and 72 °C-30 sec, and final cooling to 4 °C.

Quantification of heteroplasmy was performed by allele refractory mutation system (ARMS)-based quantitative PCR (qPCR)^32^ using an ABI PRISM 7900HT (Applied Biosystems). The reaction mixture contained 0.3 ng template DNA, GeneAce SYBR qPCR Mix α (Nippon Gene, Japan), with primers ARMS-G13513_F1WT and ARMS-G13513_R1 for wild-type mtDNA species or ARMS-G13513_F1Mut and ARMS-G13513_R1 for mutant mtDNA species (Table S1). QPCR was performed in triplicate for each DNA sample. PCR cycle conditions consisted of 95 °C-10 min, and 40 cycles of 95 °C-15 sec and 60 °C-30 sec.

### Measurement of mitochondrial DNA copy number

mtDNA copy number of fibroblasts and iPSCs was determined as described previously^25,33^, with partial modification. The reaction mixture contained 0.9 ng template DNA, GeneAce SYBR qPCR Mix α (Nippon Gene), with primers MT-CYB-F and MT-CYB-R for mtDNA, or FBXO15-F and FBXO15-R for nuclear DNA (Table S1). PCR cycle conditions consisted of 95 °C-10 min, and 40 cycles of 95 °C-15 sec and 62 °C-30 sec.

### Construction of TALEN expression plasmids

TALEN expression plasmids were constructed using a Platinum Gate TALEN Kit according to the manufacturer’s protocol. This kit was donated by Takashi Yamamoto (Addgene kit #1000000043)^11^. We adapted a simple ligation method for 4-module assembly and the Golden Gate method for final TALEN vector assembly using 4-module ligand plasmids as described in the manufacturer’s protocol of the Platinum Gate TALEN Kit. The destination vector to express pTALEN monomer with + 136/ + 63 scaffold under the control of the CAG promoter was modified to express the mpTALEN monomer. Modifications included removal of the Flag tag and nuclear localization signal (NLS), inclusion of a mitochondrial targeting sequence (MTS) derived from ATP synthase, H+ transporting, mitochondrial F1 complex, beta polypeptide (ATP5B)^7^ or cytochrome oxidase subunit 8 (COX8)^17^ and an epitope tag (Flag or HA) in the N terminus of the TALEN protein, as described in Figs 3E and S6. DNA fragments including the sequence of MTS and the epitope tag in the N terminus were inserted into destination vector digested at the Hind III and Bam HI sites using an In-Fusion HD cloning kit (Clontech).

### Single-strand annealing (SSA) assay using HEK293T cells

TALEN activity can be evaluated using a SSA assay as previously described^16^. The TALEN target sequence around m.13513G or m.13513A, named m.13513G(WT)-2 or m.13513A(MUT)-2, was amplified using XmaI-G13513GWT-2_F or XmaI-G13513A-2_F, and XmaI-G13513-2_Rch primers (Table S1) by PCR and inserted into the Xma I site between the bisected luciferase elements of the pGL4-SSA reporter plasmid^34^.

HEK293T cells were transfected with three types of plasmids, comprising TALEN plasmids, a reporter plasmid and a reference plasmid for dual-luciferase assay (pRL-CMV; Promega), by Lipofectamine LTX (Invitrogen). Dual-luciferase assays were performed at 24 hours after transfection using a Dual-Glo luciferase assay system (Promega) in an ARVO X5 luminometer (Perkin-Elmer).

### Cells: transfection and sorting

HEK293T and HeLa cells were transfected using Lipofectamine 3000 (Invitrogen) according to the manufacturer’s protocol.

MELAS-iPSCs were cultured under feeder-free conditions in accordance with a previous report^35^ for transduction of mpTALEN plasmids. After removing feeder cells, iPSCs were dissociated into single cells by incubation with 0.5× TrypLE Select (Gibco) for about 4 min at 37 °C. Single iPSCs were reseeded on dishes coated with iMatrix-511 (Nippi, Japan) and cultured using StemFit AK02N medium (Ajinomoto). One or two days later, pCAGGS-EGFP (pCAGGS^36^ vector provided by RIKEN BRC through the National Bio-Resource Project of the MEXT, Japan) and L- and R-mpTALEN plasmids were introduced into iPSCs using Lipofectamine 3000 reagent (Invitrogen) according to the manufacturer’s protocol. One or two or three days later, cells were harvested and sorted using a Moflo Astrios (Beckman Coulter) with Summit acquisition software (Beckman Coulter). A sorting gate was established based on forward and side scatter as well as the level of EGFP expression after exclusion of dead cells and debris stained with propidium iodide (PI). EGFP-positive cells were directly sorted into primate embryonic stem cell medium (ReproCell) with 10 μM Y-27632 (Wako).

### Western blotting

Transfected HEK293T cells were washed with PBS, and lysed directly with RIPA buffer containing a protease inhibitor cocktail (Roche). After sonication and centrifugation, the supernatants were separated by sodium dodecyl sulfate-polyacrylamide gel electrophoresis (SDS-PAGE) and transferred to Hybond-P (GE Healthcare). The blots were probed with an appropriate primary antibody, followed by HRP-conjugated anti-mouse IgG (GE Healthcare). The protein signals were detected using ECL prime Western blot detection reagent (GE Healthcare). The images were analyzed using an ImageQuant Las 4000 mini (GE Healthcare).

### Antibodies

Primary antibodies used were as follows: mouse anti-SSEA-4 (1:100, MAB4304, Millipore), goat anti-Nanog (1:20, AF1997, R&D Systems), rabbit anti-Oct4 (1:200, ab19857, Abcam), mouse anti-α-SMA (1:200, M0851, Dako), goat anti-Sox17 (1:200, AF1924, R&D Systems), rabbit anti-Tuj1 (1:2000, MRB-435P, Covance), mouse anti-Flag M2 (ICC, 1:500; WB, 1:100, F1804, Sigma), mouse anti-HA Tag (6E2) (ICC, 1:100; WB, 1:1000, #2367, Cell Signaling), rabbit anti-Tom20 (1:100, sc-11415, Santa Cruz), and mouse anti-GAPDH (1:1000, NB600-502, Novusbio). Alexa Fluor 488 (A11055 or A11034, Molecular Probes), Alexa Fluor 594 (A11005, Molecular Probes) and Alexa Fluor 647 (ab150075, Abcam)-conjugated secondary antibodies were used for immunofluorescent study.

### Statistical analysis

Results were analyzed using Student’s *t*-test to determine statistical significance.

Comparisons of mean among three groups were performed by one-way ANOVA followed by Tukey’s test (PRISM, GraphPad software). Differences were considered significant at *p* < 0.05. All data were expressed as mean ± SEM.

## Electronic supplementary material

Supplementary Information
