# Author Correction: TALEN-mediated shift of mitochondrial DNA heteroplasmy in MELAS-iPSCs with m.13513 G>A mutation

**DOI:** 10.1038/s41598-018-22721-y

**Published:** 2018-03-13

**Authors:** Naoki Yahata, Yuji Matsumoto, Minoru Omi, Naoki Yamamoto, Ryuji Hata

**Affiliations:** 1 Department of Anatomy I, Fujita Health University School of Medicine, Toyoake, Aichi Japan; 2 Department of Pediatrics, Fujita Health University School of Medicine, Toyoake, Aichi Japan; 3 Division of Molecularbiology, Joint Research Support Promotion Facility, Center for Research Promotion and Support, Fujita Health University, Toyoake, Aichi Japan; 4 Department of Anatomy, Ehime University Graduate School of Medicine, Shitsukawa, To-on, Ehime Japan

Correction to: *Scientific Reports* 10.1038/s41598-017-15871-y, published online 14 November 2017

This Article contains an error in the Results section. The subheading:

‘G13513A-mpTALEN increased heteroplasmy level in MELAS-iPSCs’

should read:

‘G13513A-mpTALEN decreased heteroplasmy level in MELAS-iPSCs’

