# Supplementary material for: TALEN-mediated shift of mitochondrial DNA heteroplasmy in MELAS-iPSCs with m.13513G>A mutation
Source: Sci Rep. 2017 Nov 14;7:15557. doi: 10.1038/s41598-017-15871-y (PMC5686150; doi:10.1038/s41598-017-15871-y)
Supplement: Supplementary file 1 — Supplementary Information [file 41598_2017_15871_MOESM1_ESM.pdf]

Supplementary Information

Title:

TALEN-mediated shift of mitochondrial DNA heteroplasmy in MELAS-iPSCs with m.13513G>A mutation

Authors:

Naoki Yahata, Yuji Matsumoto, Minoru Omi, Naoki Yamamoto & Ryuji Hata

Figures S1-S11

Table S1

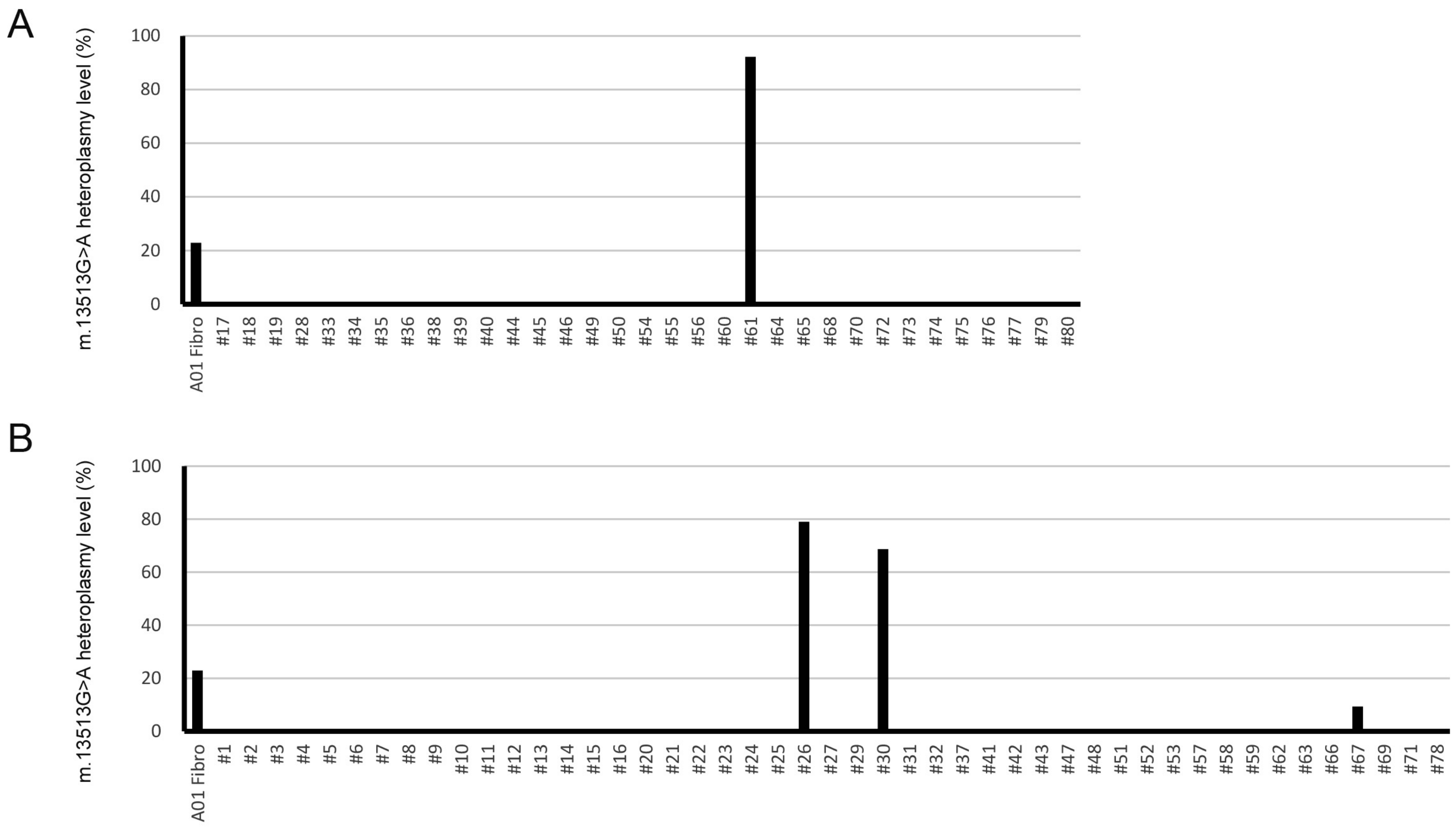

Figure S1.

m.13513G>A heteroplasmy level in all A01 MELAS-iPSC clones analyzed by ARMS-qPCR.

m.13513G>A heteroplasmy level in A01 fibroblasts and all isolated iPSC clones at passage 5 generated by Sendai virus (A) and episomal vectors (B), respectively.

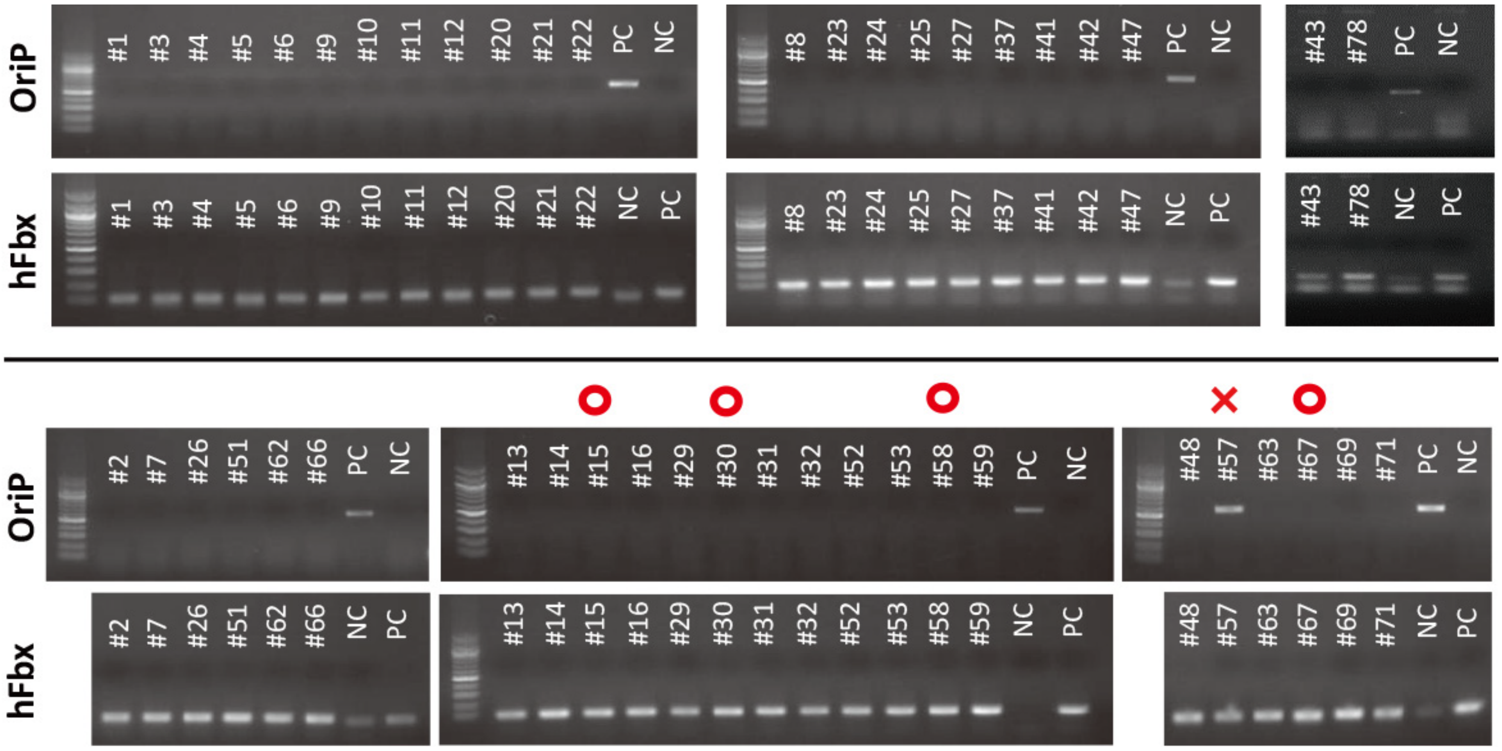

Figure S2.

Transgene expression in MELAS-iPSC clones generated by episomal vectors.

Integrated episomal vectors were detected by PCR for Ori-P cassette. Lanes labelled hFbx indicate endogenous gene as loading control. NC and PC are negative and positive controls, respectively. Circles and X mark indicate selected and excluded iPSC clones in this study, respectively.

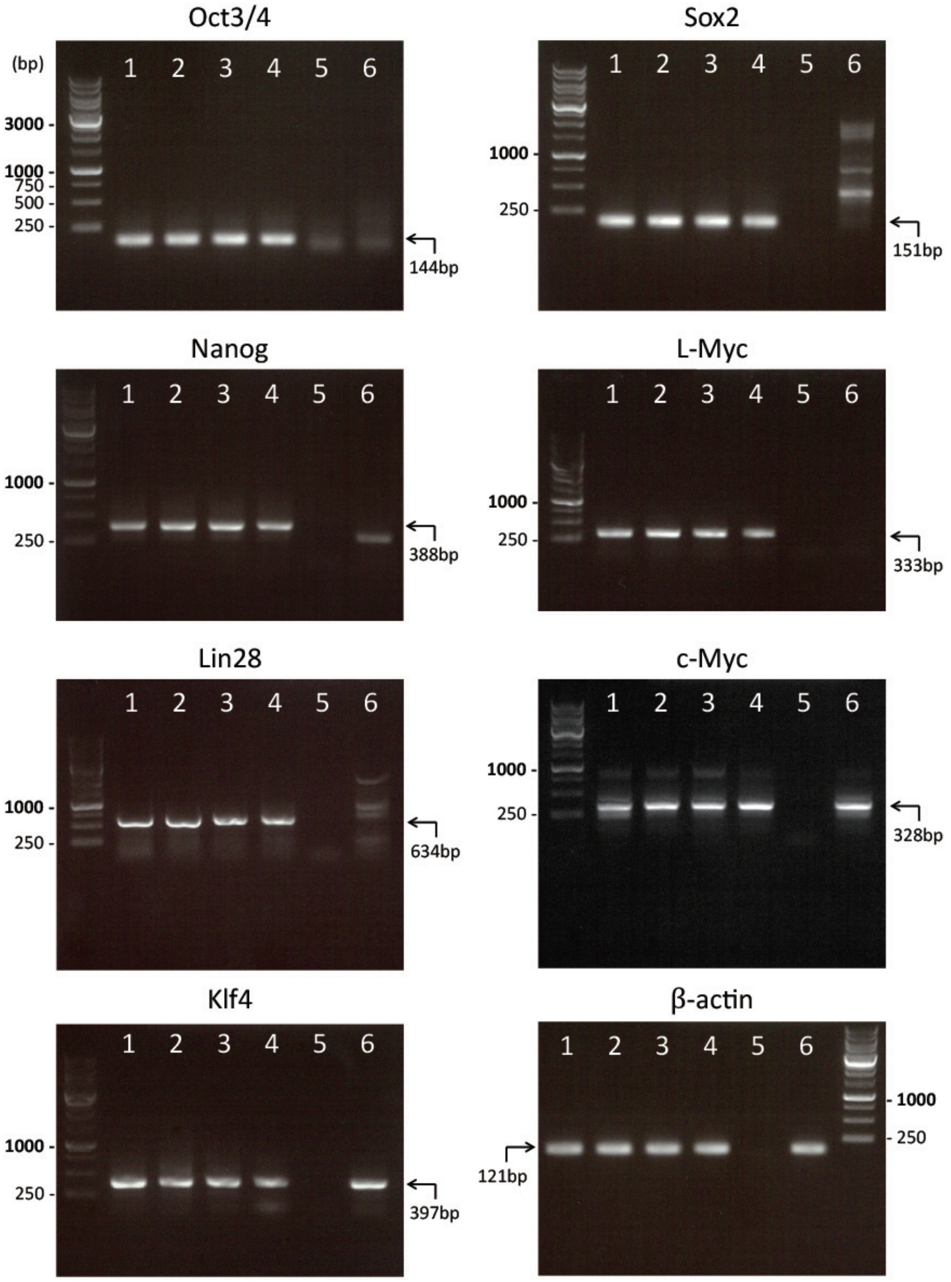

Figure S3.

Uncropped gel images for Figure 1B

Arrows indicate PCR-generated bands. Expected band size is shown on the side of the arrow.

Lane 1, A01 #15-iPSCs; Lane 2, A01 #58-iPSCs; Lane 3, A01 #30-iPSCs;

Lane 4, A01 #67-iPSCs; Lane 5, Water; Lane 6, A01 fibroblasts.

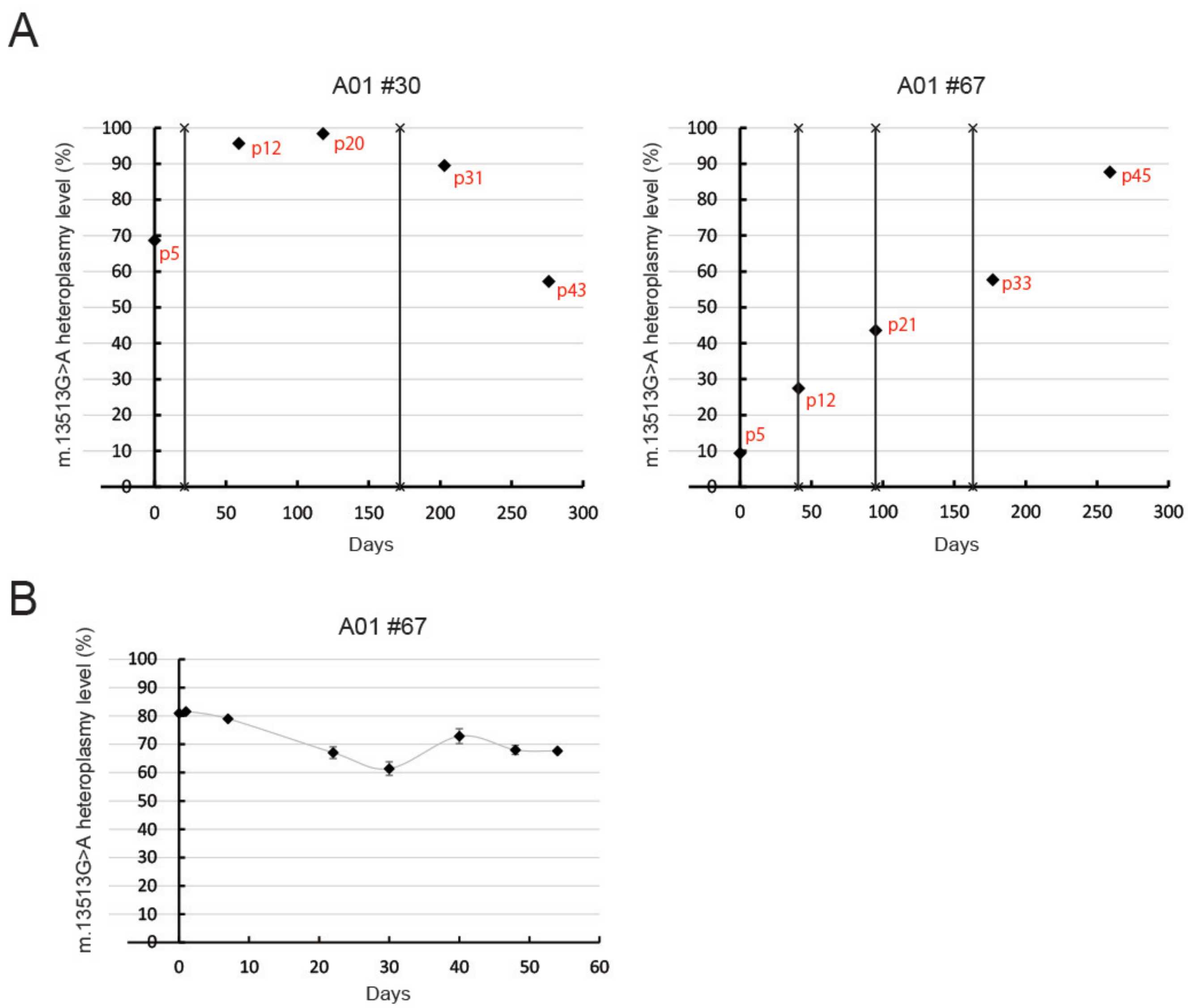

Figure S4.

Genetic fluctuation of m.13513G>A heteroplasmy level in A01 MELAS-iPSCs during their cultivation.

(A) Long-term cultivation of A01 #30-iPSCs (left) and A01 #67-iPSCs (right) on feeder layers. Red letters beside diamonds indicate passage numbers. iPSCs were re-cultured after freezing and thawing at the time points indicated by vertical lines. (B) Continuous culture of A01 #67-iPSCs under feeder-free condition. Data are expressed as mean ± SEM (n = 3).

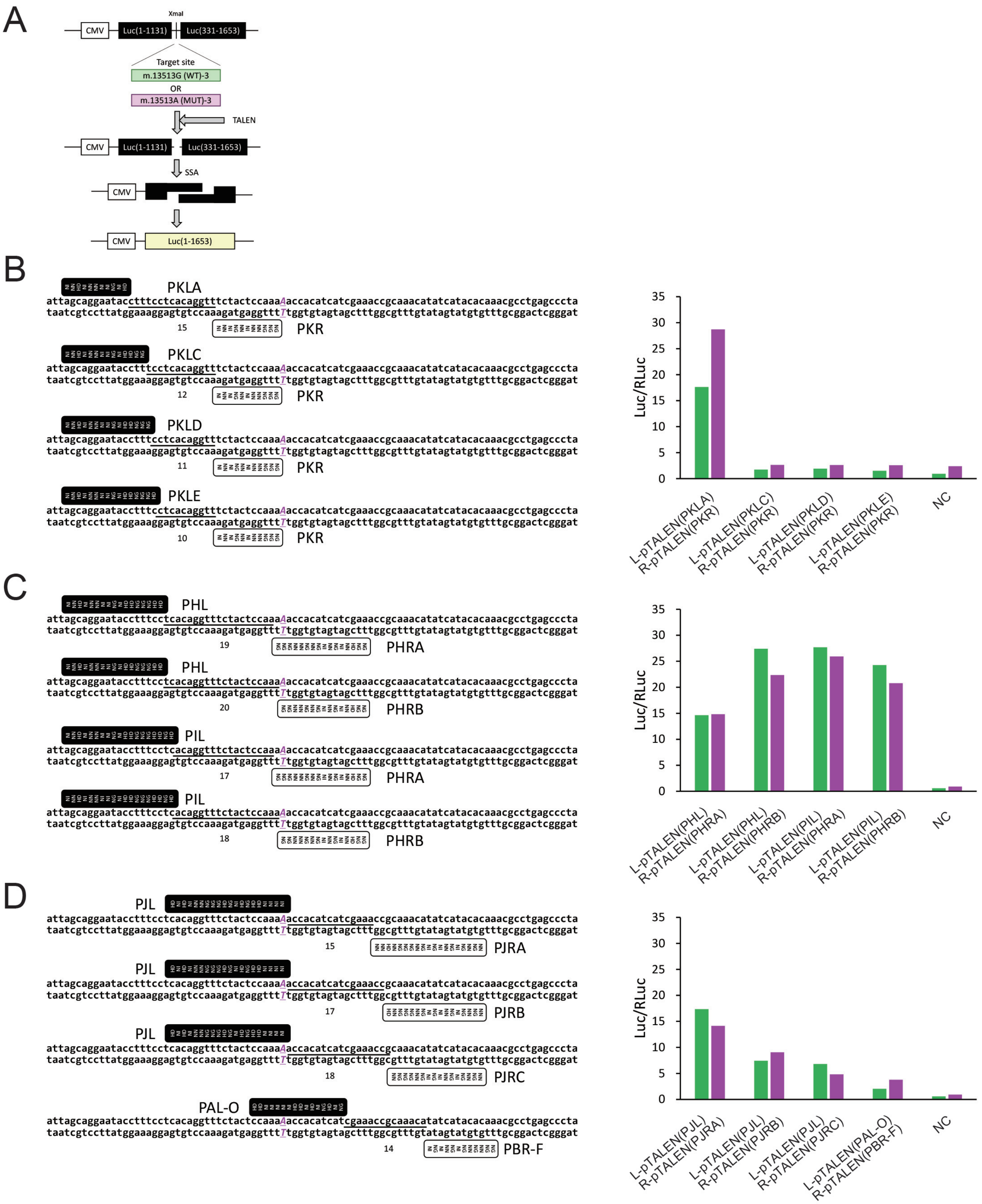

Figure S5.

Functional evaluation of engineered of G13513A-pTALENs by SSA assay.

(A) Scheme of SSA assay. The TALEN target sequence around m.13513G or m.13513A, named m.13513G(WT)-3 or m.13513A (MUT)-3, was amplified using XmaI-G13513GWT-3_F or XmaI-G13513A-3_F, and XmaI-G13513_R primers (Table S1) by PCR and inserted into the Xma I site between the bisected luciferase elements of the pGL4-SSA reporter plasmid. (B, C and D) Left, Schematic design of G13513A-pTALENs used in each assay. Black and white boxes indicate RVDs of L-pTALEN and R-pTALEN, respectively. Black bar and number indicate spacer region and spacer length (bp), respectively. Letters beside box indicate TALE’ s name. Right, evaluation of SSA activity (Luc/RLuc) of pTALEN pairs (n = 1). Green and purple bars reflect cleaving activity against m.13513G(WT)-3 and m.13513A(MUT)-3, respectively. NC, negative control.

L-mpTALEN(PKLB)

MTS of ATP5B

MLGFVGRVAAAPASGALRRLTPSASLPPAQLLLR
AAPTAVHPVRDYAAQDYKDDDDK Flag Tag
VDLRTLGYSQQQQEKIKPKVRSTVAQHHEALVGH
GFTHAHIVALSQHPAALGTVAVKYQDMIAALPEA
THEAIVGVGKQWSGARALEALLTVAGELRGPPLQ
LDTGQLLKIAKRGGVTAVEAVHAWRNALTGAPLN
LTPDQVVAIASNIGGKQALETVQRLLPVLCQDHG
LTPEQVVAIASNNGGKQALETVQRLLPVLCQAHG
LTPDQVVAIASHDGGKQALETVQRLLPVLCQAHG
LTPAQVVAIASNIGGKQALETVQRLLPVLCQDHG
LTPDQVVAIASNNGGKQALETVQRLLPVLCQDHG
LTPEQVVAIASNNGGKQALETVQRLLPVLCQAHG
LTPDQVVAIASNIGGKQALETVQRLLPVLCQAHG
LTPAQVVAIASNIGGKQALETVQRLLPVLCQDHG
LTPDQVVAIASNGGGKQALETVQRLLPVLCQDHG
LTPEQVVAIASNIGGKQALETVQRLLPVLCQAHG
LTPDQVVAIASHDGGKQALETVQRLLPVLCQAHG
LTPAQVVAIASHDGGKQALETVQRLLPVLCQDHG
LTPEQVVAIASNGGGRPALE
SIVAQLSRPDPALAALTNDHLVALACLGGRPALD
AVKKGLPHAPALIKRTNRRIPERTSHRVA
GSQLVKSELEEKKSELRHKLKYVPHEYIELIEIA
RNSTQDRILEMKVMEFFMKVYGYRGKHLGGSRKP
DGAIYTVGSPIDYGVIVDTKAYSGGYNLPIGQAD
EMQRYVEENQTRNKHINPNEWWKVYPSSVTEFKF
LFVSGHFKGNYKAQLTRLNHITNCNGAVLSVEEL
LIGGEMIKAGTLTLEEVRRKFNNGEINFRS*

Fok I nuclease domain

R-mpTALEN(PKR)

MTS of COX8 HA Tag

MSVLTPLLLRGLTGSARRLPVPRAAAAYPYDVPD
YA
VDLRTLGYSQQQQEKIKPKVRSTVAQHHEALVGH
GFTHAHIVALSQHPAALGTVAVKYQDMIAALPEA
THEAIVGVGKQWSGARALEALLTVAGELRGPPLQ
LDTGQLLKIAKRGGVTAVEAVHAWHNALTGAPLN
LTPDQVVAIASNGGGKQALETVQRLLPVLCQDHG
LTPEQVVAIASNGGGKQALETVQRLLPVLCQAHG
LTPDQVVAIASNGGGKQALETVQRLLPVLCQAHG
LTPAQVVAIASNNGGKQALETVQRLLPVLCQDHG
LTPDQVVAIASNNGGKQALETVQRLLPVLCQDHG
LTPEQVVAIASNIGGKQALETVQRLLPVLCQAHG
LTPDQVVAIASNNGGKQALETVQRLLPVLCQAHG
LTPAQVVAIASNGGGKQALETVQRLLPVLCQDHG
LTPDQVVAIASNIGGKQALETVQRLLPVLCQDHG
LTPEQVVAIASNNGGKQALETVQRLLPVLCQAHG
LTPEQVVAIASNIGGRPALE
SIVAQLSRPDPALAALTNDHLVALACLGGRPALD
AVKKGLPHAPALIKRTNRRIPERTSHRVA
GSQLVKSELEEKKSELRHKLKYVPHEYIELIEIA
RNSTQDRILEMKVMEFFMKVYGYRGKHLGGSRKP
DGAIYTVGSPIDYGVIVDTKAYSGGYNLPIGQAD
EMQRYVEENQTRNKHINPNEWWKVYPSSVTEFKF
LFVSGHFKGNYKAQLTRLNHITNCNGAVLSVEEL
LIGGEMIKAGTLTLEEVRRKFNNGEINFRS*

Fok I nuclease domain

Figure S6.

Amino-acid sequences of L-mpTALEN(PKLB) and R-mpTALEN(PKR).

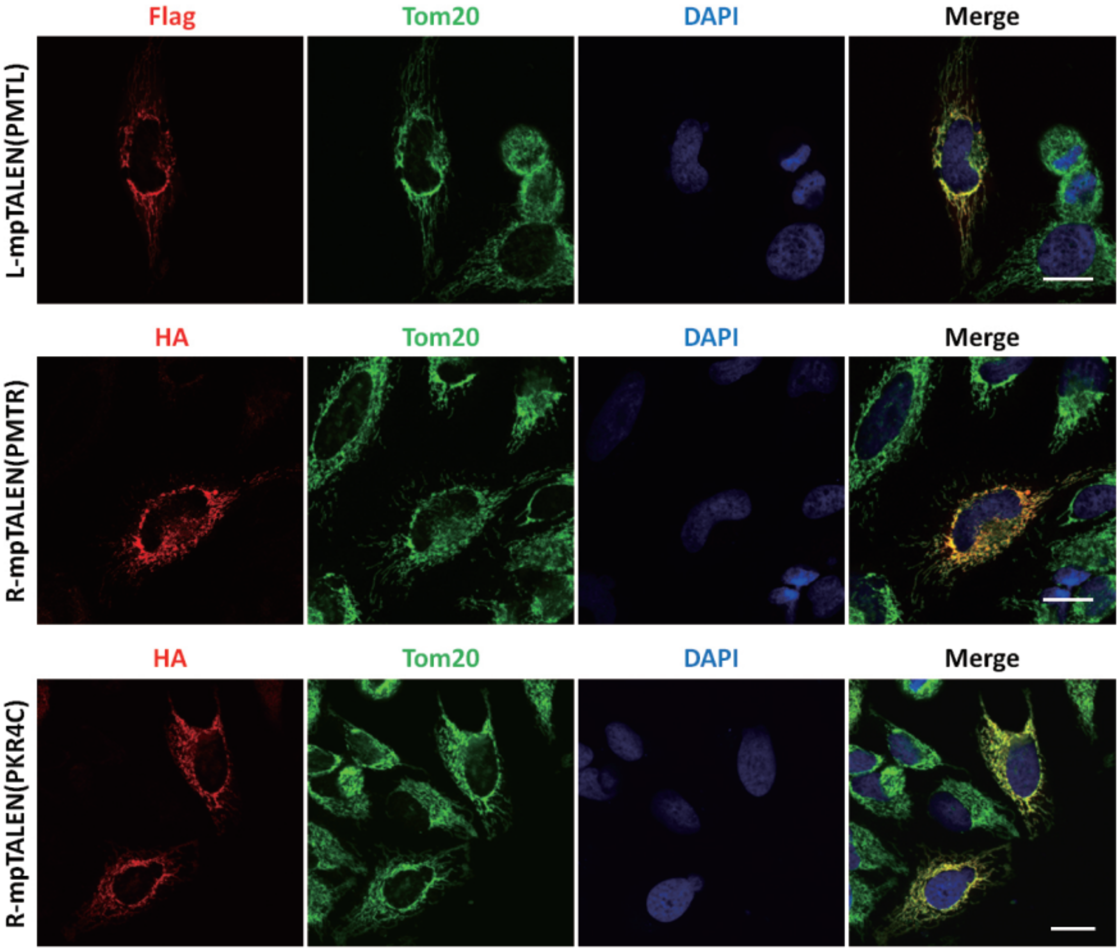

Figure S7.

Mitochondrial localization of mpTALENs analyzed by immunocytochemical analysis.

mpTALEN monomer was transiently expressed in HeLa cells. Two days after transfection, L-mpTALEN(PMTL) (top), R-mpTALEN (PMTR) (middle), or R-mpTALEN(PKR4C) (bottom) was stained using anti-Flag or anti-HA antibodies (red). Mitochondria were stained using anti-TOM20 antibodies (green). Nuclei were stained with DAPI (blue). Scale bar, 20 μm.

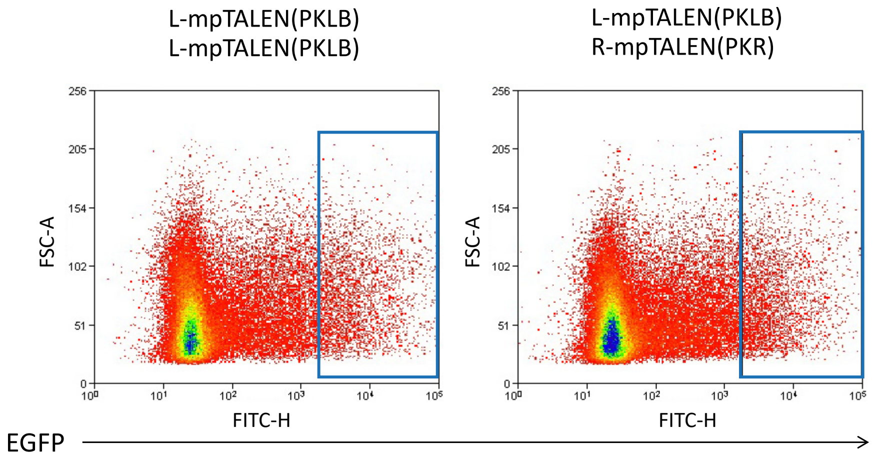

Figure S8.

Dot plots of EGFP fluorescent signal versus FSC-A in cell sorting.

Cell sorting was performed according to the experimental scheme shown in Figure 4A. Two days after transfection of mpTALEN and EGFP plasmids, EGFP-positive cells selected by the blue square gate were sorted. Right, cell sample transfected with L-mp-TALEN(PKLB) and R-mpTALEN(PKR) plasmids; Left, cell sample transfected with twice the amount of L-mpTALEN(PKLB) plasmid. FSC-A, forward scatter-area; FITC-H, fluorescein isothiocyanate-height.

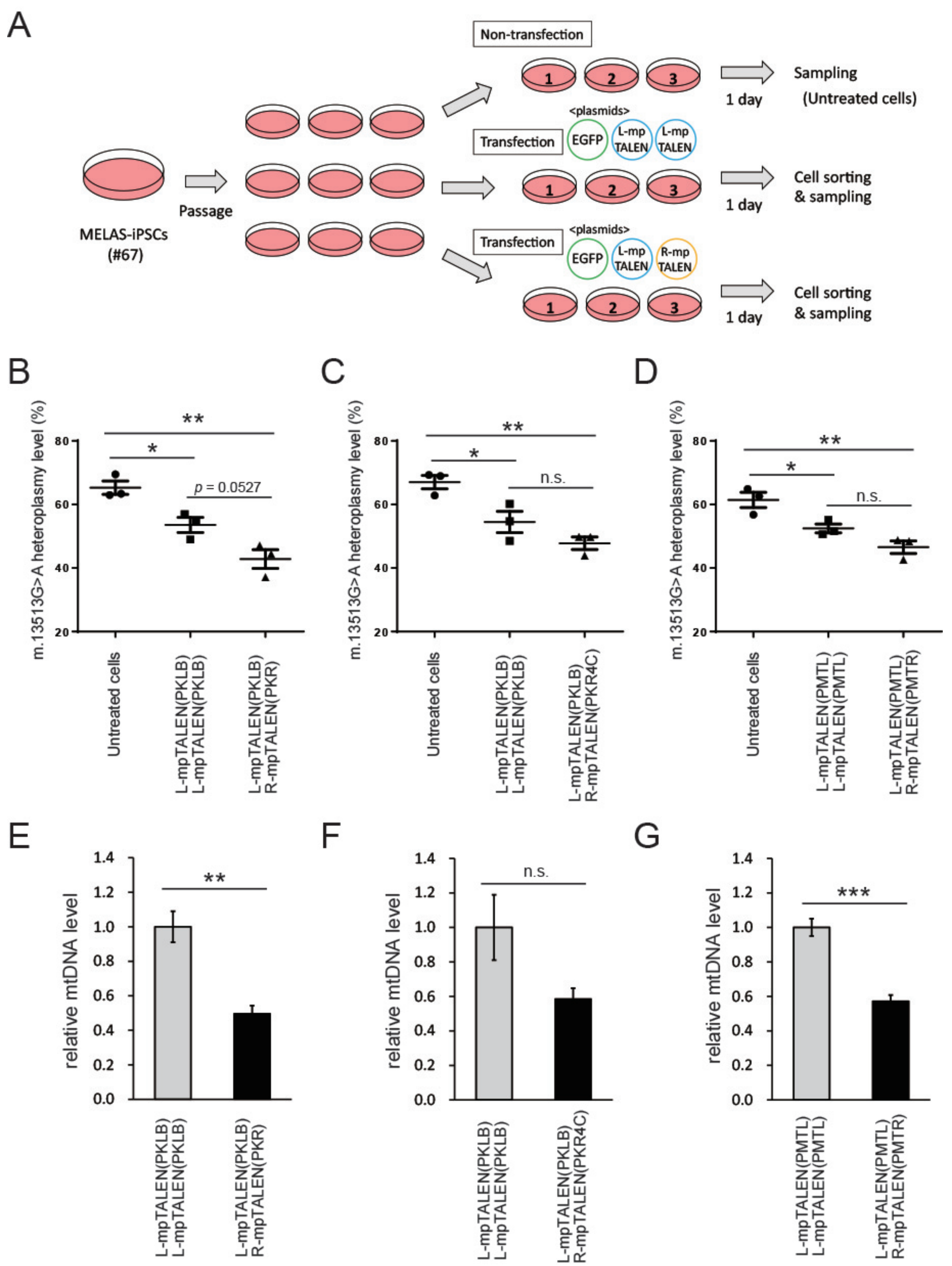

Figure S9.

Effects of three types of G13513A-mpTALEN pairs on heteroplasmy level in A01 #67-iPSCs at day 1 after transfection.

(A) Experimental scheme. MELAS-iPSCs (#67) subcultured at the same time were transfected with plasmids coding L-mpTALEN and R-mpTALEN and EGFP (n = 3). EGFP-positive and live cells sorted at day 1 after transfection were compared with sorted cells transfected with twice the amount of plasmid coding L-mpTALEN, and untreated cells. (B, E) L-mpTALEN(PKLB)/R-mp-TALEN(PKR) pair; (C, F) L-mpTALEN(PKLB)/R-mpTALEN(PKR4C) pair; (D, G) L-mpTALEN(PMTL)/R-mpTALEN(PMTR). (B, C and D) m.13513G>A heteroplasmy level was analyzed by ARMS-qPCR. Data are expressed as mean ± SEM (n = 3). *$p < 0.05$, **$p < 0.01$, Tukey' s test. (E, F and G) MtDNA copy numbers of L-mpTALEN/R-mpTALEN are represented relative to those of L-mpTALEN/L-mpTALEN. Data are expressed as mean ± SEM (n = 3). **$p < 0.01$, ***$p < 0.005$, Student' s *t*-test.

<Experiment 2>

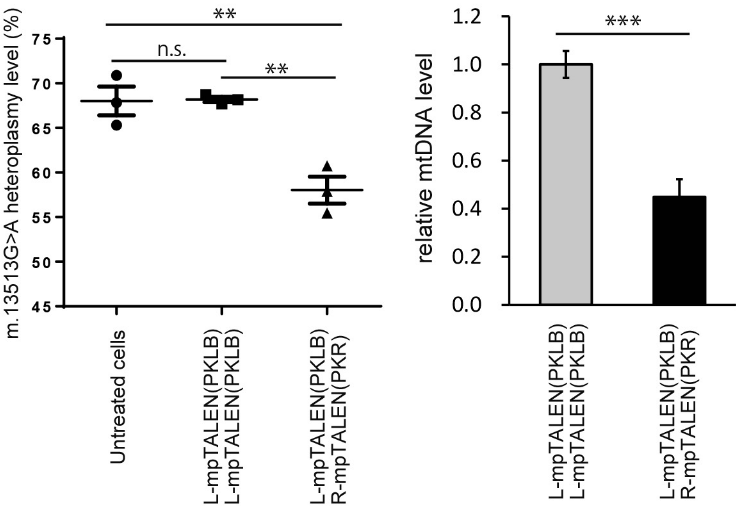

<Experiment 3>

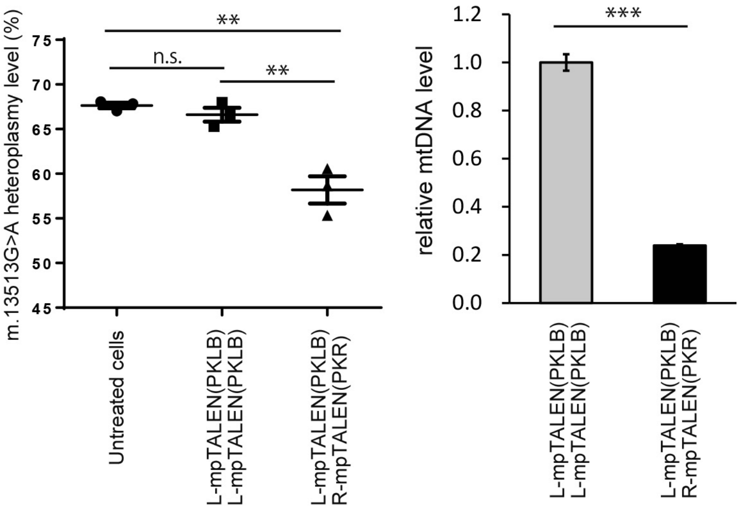

Figure S10.

Decrease of m.13513G>A heteroplasmy level in MELAS-iPSCs by G13513A-mpTALEN.

According to the scheme in Figure 4A, an additional two independent experiments were performed (Experiments 2 and 3). Left, m.13513G>A heteroplasmy level was analyzed by ARMS-qPCR. Data are expressed as mean ± SEM (n = 3). **$p < 0.01$, Tukey’ s test. Right, MtDNA copy number of L-mpTALEN(PKLB)/R-mpTALEN(PKR) is represented relative to that of L-mpTALEN (PKLB)/L-mpTALEN(PKLB). Data are expressed as mean ± SEM (n = 3). ***$p < 0.005$, Student’ s *t*-test.

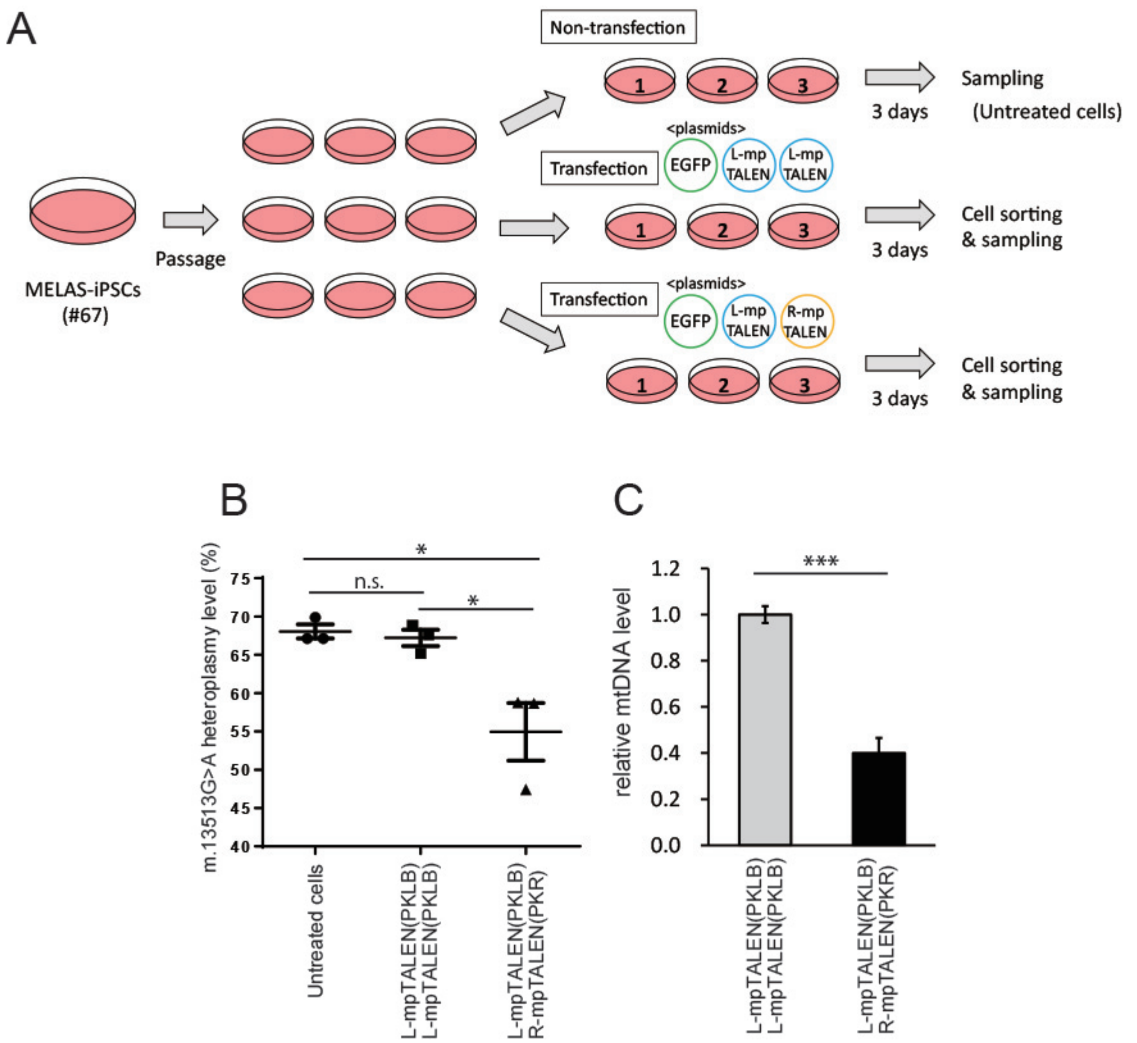

Figure S11.

Effect of L-mpTALEN(PKLB)/R-mpTALEN(PKR) pair on heteroplasmy level in A01 #67-iPSCs at day 3 after transfection.

(A) Experimental scheme. MELAS-iPSCs (#67) subcultured at the same time were transfected with plasmids coding L-mpTALEN (PKLB) and R-mpTALEN(PKR) and EGFP (n = 3). EGFP-positive and live cells sorted at day 3 after transfection were compared with sorted cells transfected with twice the amount of plasmid coding L-mpTALEN(PKLB), and untreated cells. (B) m.13513G>A heteroplasmy level was analyzed by ARMS-qPCR. Data are expressed as mean ± SEM (n = 3). *$p < 0.05$, Tukey' s test. (C) MtDNA copy numbers of L-mpTALEN(PKLB)/R-mpTALEN(PKR) are represented relative to those of L-mpTALEN(PKLB)/L-mp-TALEN(PKLB). Data are expressed as mean ± SEM (n = 3). ***$p < 0.005$, Student' s *t*-test.

Table S1: Primer list

Name	Sequence	Name	Sequence
rt-Oct3/4-F	GACAGGGGGAGGGGAGGAGCTAGG	rt-Oct3/4-R	CTTCCCTCCAACCAGTTGCCCCAAAC
rt-Sox2-F	GGGAAATGGGAGGGGTGCAAAAGAGG	rt-Sox2-R	TTGCGTGAGTGTGGATGGGATTGGTG
rt-Klf4-F	ACGATCGTGGCCCCGGAAAAGGACC	rt-Klf4-R	TGATTGTAGTGCTTTCTGGCTGGGCTCC
rt-L-Myc-F	GTGAGTCCCCCACCTGTAGA	rt-L-Myc-R	TTAGTAGCCAGTGAGGTATGCAATTC
rt c-Myc-F	GCGTCCTGGGAAGGGAGATCCGGAGC	rt c-Myc-R	TTGAGGGGCATCGTCGCGGGAGGCTG
rt-Lin28-F	CACCATGGGCTCCGTGTCCAACCAGCAG	rt-Lin28-R	TCAATTCTGTGCCTCCGGGAGCAGGGTAGG
rt-Nanog-F	CAGCCCCGATTCTTCCACCAGTCCC	rt-Nanog-R	CGGAAGATTCCCAGTCGGGTTCACC
ACTB-F	CCAACCGCGAGAAGATGA	ACTB-R	TCCATCACGATGCCAGTG
Mito-3F	TCATTTTTATTGCCACAACTAACCTCCTCGGACTC	Mito-3R	CGTGATGTCTTATTTAAGGGGAACGTGTGGGCTAT
ARMS-G13513_F1WT	CTCACAGGTTTCTACTCCAATG	ARMS-G13513_R1	GACCTGTTAGGGTGAGAAGAA
ARMS-G13513_F1MUT	CTCACAGGTTTCTACTCCAATA		
G13513A.02F	CCTCACAGGTTTCTACTCCGAA	G13513A.02R	CGAGTGCTATAGGCGCTT
MT-CYTB-F	TGCAACTATAGCAACAGCCTTCA	MT-CYTB-R	GAACTAGGTCTGTCCCAATGTATGG
FBXO15-F	GCCAGGAGGTCTTCGCTGTA	FBXO15-R	AATGCACGGCTAGGGTCAAA
pEP4-SF1	TTCCACGAGGGTAGTGAACC	pEP4-SR1	TCGGGGGTGTTAGAGACAAC
hFbx15-2F	GCCAGGAGGTCTTCGCTGTA	hFbx15-2R	AATGCACGGCTAGGGTCAAA
XmaI-G13513GWT-2_F	TTACCCGGGTCGATGATGTGGTCTTTGGA	XmaI-G13513-2_Rch	TAGCCCGGGCTCACCATTGGCAGCCTAGC
XmaI-G13513A-2_F	TTACCCGGGTCGATGATGTGGTTTTTGGA		
XmaI-G13513GWT-3_F	TTACCCGGGATTAGCAGGAATACCTTTCCTCACAGGTTTCTACTCCAAAG	XmaI-G13513_R	TAACCCGGGTAGGGCTCAGGCGTTTGTGT
XmaI-G13513A-3_F	TTACCCGGGATTAGCAGGAATACCTTTCCTCACAGGTTTCTACTCCAAAA		
